# Positron Emission Tomography (PET) radiotracers in oncology – utility of 18F-Fluoro-deoxy-glucose (FDG)-PET in the management of patients with non-small-cell lung cancer (NSCLC)

**DOI:** 10.1186/1756-9966-27-52

**Published:** 2008-10-17

**Authors:** Evelina Miele, Gian Paolo Spinelli, Federica Tomao, Angelo Zullo, Filippo De Marinis, Giulia Pasciuti, Luigi Rossi, Federica Zoratto, Silverio Tomao

**Affiliations:** 1 Department of Experimental Medicine University of Rome "Sapienza" viale Regina Elena 324, Rome, Italy; 2 Operative Unit of Medical Oncology, S. Maria Goretti Hospital, University of Rome "Sapienza" – Latina-via Canova, Latina, Italy; 3 Gastroenterology and Digestive Endoscopy, "Nuovo Regina Margherita" Hospital, Rome 00153, Italy; 4 Thoracic Oncology Unit I, Department of Lung Diseases, San Camillo and Forlanini Hospitals, Rome, Italy

## Abstract

PET (Positron Emission Tomography) is a nuclear medicine imaging method, frequently used in oncology during the last years. It is a non-invasive technique that provides quantitative in vivo assessment of physiological and biological phenomena. PET has found its application in common practice for the management of various cancers.

Lung cancer is the most common cause of death for cancer in western countries.

This review focuses on radiotracers used for PET scan with particular attention to Non Small Cell Lung Cancer diagnosis, staging, response to treatment and follow-up

## Introduction

PET scan represents one of the most sophisticated nuclear medicine techniques of the last years. It was initially used to study the brain and the heart, but today it is used mainly in oncology. PET scanning is a non-invasive imaging method that differs from others because it observes "in vivo" metabolic activity using radio-isotopes with specific tissutal tropism. More in detail PET scans necessitate the injection of a small quantity of biologically important material like glucose or oxygen which have labelled with radio-nuclides such as carbon-11, nitrogen-13, oxygen-15 and fluoride-18.

All the used isotopes are radioactive with a rapid time of decaying by positron emission: carbon-11 or 11C is a radioactive isotope of carbon with a half-life in the order of twenty minutes. Nitrogen-13 or 13N is an isotope of nitrogen with a half life of approximately ten minutes. Oxygen-15 or 15O is an isotope of oxygen having a half life of about two minutes.

The most commonly used isotope in PET scans is fluorine-18. It is a fluorine isotope with a half life of approximately 110 minutes. This tracer is very useful because of its long half life and because it decays by emitting positrons having the lowest positron energy which contributes to a high-resolution imaging acquire.

Most articles have considered the utility of FDG (fluorine-18 combined with deoxy-glucose) which is the most used radiotracer in clinical practice. Actually, a number of new compounds with promising prospective for PET imaging are also available to gain information about specific cellular and molecular tumour pathways.

In the second part of this review a wide panoramic on the use of this technique will be conducted regarding an important oncologic pathology, both in term of incidence and mortality: lung cancer.

### 18F-FGD and Alternative tracers to 18F-FDG in oncology and other diseases

#### 18F-FDG

**Figure 1:**
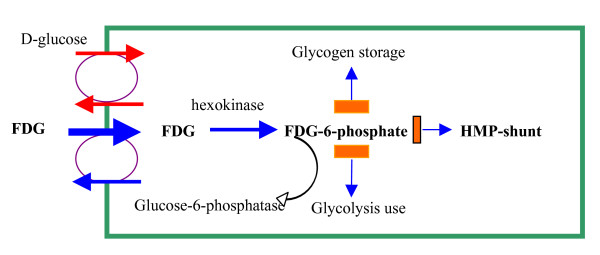
**Mechanism of Glucose trapping in FDG-PET**[**98**].

FDG is a glucose analogue extensively used in oncology for staging, restaging and recently for the evaluation of tumour response to treatment [1]. Cancer cells demonstrate up regulation of glucose metabolism: uptake of glucose or glucose-analogues, as deoxy-glucose is increased. Labeling deoxy-glucose with the positron emitting radionuclide 18F to form 18F-FDG renders these cells detectable using PET. In detail, 18-FDG is transported into the cells by the same carrier as glucose, but at a much higher rate. Then it is phosphorylated to FDG-6-phosphate (FDG-6-P) by the action of hexokinase or glucokinase [2]. This substance does not enter the standard metabolic pathways because of the presence of fluorine at the C-2 position of the ring instead of the hydroxyl group in glucose and can leave the cell only slowly by the action of glucose-6-phosphatase. So it is trapped and accumulated in the neoplastic cells. This 'metabolic trapping' of FDG-6-P forms the basis of the analysis of PET data. (Fig 1)

The basis for using FDG in oncology was demonstrated by Warburg who observed an increase in glycolytic activity in cancer cells, under both aerobic and anaerobic conditions [3-5]. Moreover neoplastic transformation often determines an increase in the activity of glycolytic enzymes (e.g. hexokinase) and in glucose transporters (e.g. GLUT1) [6]. Cell mass influences this glycolytic activity, while cell proliferation affects the increase in glucose transport. Otherwise this condition is not specific to malignant tumors; in fact accumulation of glucose can also be present in benign pathology and inflammatory disease, where activated inflammatory cells or macrophages also use glucose as energy. Clinically inflammatory foci, sarcoidosis, and active tuberculosis are often shown as FDG positive lesions [7,8]. On the other hand some malignant cells such as carcinoid tumors and bronchioalveolar lung carcinoma are FDG-negative [9]. Moreover in hepatocellular carcinoma, highly differentiated cancer cells are FDG-negative and poorly differentiated cancer cells are FDG-positive; the FDG-positive cells indicate poor prognosis, reflecting the histological cell type.[10]

**Figure 2:**
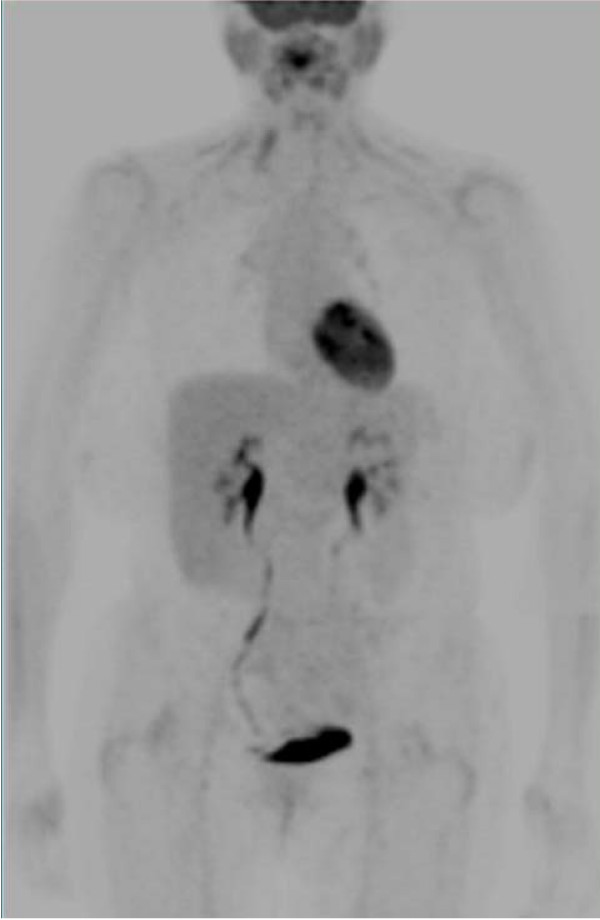
Normal PET imaging.

The clinical utility of PET can be limited by FDG distribution in some normal tissues, which can result in a low or decreased tumor to background ratio (Fig 2). In fact, the normal brain has a high glucose uptake, while in most brain tumors the uptake of FDG is similar or lower than in the normal tissue. The excretion of FDG makes urine extremely radioactive. Although bladder and prostate tumors are FDG-avid cancers this condition can create problems in the diagnosis of these neoplasms [11].

While traditional radiological imaging techniques (e.g. Computed Tomography-CT-scan, Magnetic Resonance Imaging-MRI) make available structural information and identify disease states on the basis of cross anatomical changes, FDG PET imaging provides information on the biochemical processes which may precede cross anatomic changes. [12]. The limited spatial resolution of PET due to a lack of anatomic information can be overcome by combining morphologic CT and functional PET data.

Thus functional scans obtained with FDG PET are not only complementary to those obtained with conventional modalities but also may be more sensitive because alterations in tissue metabolism generally anticipate anatomic alterations.

According to EORTC guidelines for better evaluation of lesions, PET must be combined with Computed Tomography, but response criteria are still not available [13].

To date newer PET/CT protocols have been developed, but there is no consensus about them or their standardization [14]. This remains a fundamental objective to better establish the efficacy of PET/CT for specific clinical application. In fact, high quality contrast enhanced CT scans, obtained in parallel with PET images, can contribute to diagnosis, anatomic correlation and attenuation correction of PET scans.

To correctly determine FDG PET activity it has been introduced the Maximum Standardized Uptake Value (SUV max). It is defined as maximum tumour concentration of FDG divided by the injected dose, corrected for the body weight of the patient: (SUV max = maximum activity concentration [injected dose/body weight]). It represents the metabolic activity for the tumor compared with that in surrounding tissue, corrected for injected dose and patient weight.

#### 18F-Fluoro-L-dihydrophenylamine

Among the newest studied tracers there is^18 ^F-Fluoro-L-dihydroxyphenylamine (^18^F-fluoro-L-DOPA). It is used to evaluate the in vivo activity of aromatic l-amino acid decarboxylase of dopaminergic system. For these characteristics this compound has been used in clinical practice for parkinsonisms diagnosis and to investigate subclinical lesions of the substantia nigra in Parkinson disease related disorders.[15] Moreover it has been recently found application for detection of neuroendocrine tumours (NETs), that are capable to accumulate and decarboxylate amine precursors resulting in a high uptake in PET scans with^18^F-fluoro-L-DOPA [1].

#### Somatostatin tracers

Somatostatin based radiotracers (analogues of somatostatin labelled with radioisotopes) are useful for diagnosis in patients with cancers (Neuroendocrine Tumours-NETs) which express the somatostatin receptor 2 (SSTR2). They are able to orient treatment on the basis of the quality and quantity of tracer uptake. In Fact well differentiated NET tumours do not necessarily shown an increased glucose metabolism, while they can be treated with new target drugs (Sunitinib) in addition to somatostatin analogues. On the other hand poor differentiated NETs and with high proliferation index are more commonly treated with cytotoxic drugs. [16,17]

#### 11C-Choline

Choline is a precursor of biosynthesis of an essential element of phospholipids of the cell membrane (phosphatidil – choline). In neoplastic tissue there is an elevated level of phosphatidil-choline and an up-regulation of the enzyme that catalyzes the phosphorilation of choline [18,19].

Thus a new PET radiotracer (^11^C-Choline) has been developed and is useful mostly to detect well differentiated tumours with low glucose uptake [20,21]. In clinical research PET choline is mostly studied in prostate cancer, also because of the low sensitivity and specificity of ^18^F-FDG. In particular the usefulness of this test is related principally to the detection of local tumour recurrence after radical prostatectomy or distant metastatic disease [22] or to assess the response to hormonal therapy in patients with androgen-dependent tumours or chemotherapy for patients with androgen – independent tumours [23].

#### 18F-16β-Fluoro-5-dihydrotestosterone

^18^F-16 β-Fluoro-5α-dyhidrotestosterone (FDHT), a structural analogue of 5-α-dyhidrotestosterone, can be useful to detect metastatic and recurrent prostate cancer lesions, binding affinity and selectivity for androgen receptors (ARs) [24].

#### 18F-FLT

^18^F-3-Fluoro-3-deoxy-thymidine (18-F-FLT) is a pyrimidine analogue that reveals the activities of thymidine-kinase-1 during the phase S of mitoses [25]. First it was considered a promising radiotracer for its biological characteristics, but several studies have analyzed the role of this molecule in different tumors showing a lower uptake of 18-F-FLT than 18-F-FDG, with the exception of brain tumors. [26-29]

#### 11C-Acetate

11C-Acetate is a metabolic substrate for synthesis of cholesterol and lipids. [30] This compound has not a renal clearance and initially was considered to be able to play an important role for imaging of prostate and kidney tumors. Following studies did not confirm the primitive hypothesis showing that acetate accumulation in renal cell carcinoma was lower or similar to normal kidney parenchyma, [31] while it remains promising in prostate cancer and hepatocellular carcinoma. [32-34]

#### 18F-Fluoride. 11C-Methionine

Fluorine-18-Fluoride is a PET tracer with elevated utility for detection of bone metastases in various tumors [35,36]. In fact fluoride ion is switched with hydroxyl group in the bone crystals forming fluoroapatite: where the turnover is greatest there are high deposits. An increased ^18^F-Fluoride PET uptake in bone lesions reveals both an increased bone turnover and blood flow.

Another studied tracer is represented by 11C-Methionine. Methionine is an important amino acid for protein synthesis process. The role of this tracer has been evaluated in various trials, mostly in brain tumors [37]. because it does not accumulate in normal brain tissue while FDG can not be valuable for the high glucose activity of normal brain. Other studied fields of application of 11C-Methionine have been head and neck squamous cell carcinoma (HNSCC) and prostate cancer, with minor oncological relevance [38,39].

### Utility of FDG PET in NSCLC

#### Non Small Cell Lung Cancer

Lung Cancer is one of the most important causes of tumour-related deaths in industrialized countries. NSCLC treatment includes surgery, radiation therapy, chemotherapy and molecular therapy. The choice of treatment alone or in combination is based on clinical and pathological tumour stage.

PET scan plays an important role for tumour diagnosis and staging, providing functional information simultaneous to anatomical details when PET is combined with computed tomography (CT)

#### Initial Staging

The mainly point to establish a correct staging of NSCLC is to identify patients candidates to surgery versus those ones who are inoperable but can obtain benefits from chemotherapy, radiotherapy or both.

In detail, patients with N0 – N1 disease (no metastatic lymph nodes or only intrapulmonary/hilar nodes) are generally candidates for surgical resection. On the contrary patients with N2 disease (ipsilateral mediastinal lymph nodes metastases) could gain benefit from a combination of local and systemic treatments. Patients with N3 disease (contra lateral mediastinal lymph nodes metastases) are considered for non operability. [40]

Thus, an accurate loco regional lymph nodes assessment is mandatory to choose the best treatment options. This could be done either with invasive techniques (first of all mediastinoscopy) or with non-invasive exams such as CT or FDG PET.

Among the invasive techniques, endobronchial ultrasound guided-fine needle aspiration (EUS-FNA) deserves a particular discussion. This new technique is now in development in many centers. First data confirmed a sensibility and specificity comparable to mediastinoscopy for staging malignant nodes in NSCLC [41]. In a recent meta-analysis Micames et al., analyzing 18 eligible studies, in order to estimate the diagnostic accuracy of EUS-FNA for staging mediastinal lymph nodes (N2/N3 disease) in patients with lung cancer, have concluded that EUS-FNA is a safe modality for the invasive staging of lung cancer. Moreover it is highly sensitive when used to confirm metastasis to mediastinal lymph nodes seen on CT scans and has the potential to prevent unnecessary surgery in a large proportion of cases [42].

On the other hand it has been shown the relative frequency of metastatic N2 disease in the posterior mediastinal lymph nodes which are not accessible via mediastinoscopy but are easily and accurately sampled by EUS-FNA [43]. Thus some argue that both mediastinoscopy and EUS-FNA should be performed routinely in all patients prior to resection.[44]

The increasing availability of FGD PET offers a noninvasive, accurate alternative for staging the mediastinum. Whether or not FDG-PET should be used as a routine procedure in mediastinal lymph node staging, replacing mediastinoscopy, is still a matter of debate [45].

American College of Chest Physicians guidelines [46] recommend FDG PET for non invasive staging due to the low sensitivity and specificity of the commonly used CT in the identification of node involvement. A large number of studies on PET accuracy, varying in quality and design, have evaluated its role in mediastinal lymph nodes staging using surgery (mediastinoscopy and/or thoracotomy with mediastinal lymph node dissection) as the gold standard of comparison [47-56]. They have been previously summarized in six meta-analyses (Tab 1) and convincingly demonstrated that PET is an imaging technique, superior to CT, for mediastinal lymph node staging terms of accuracy [57-62]. In fact CT scanning is mainly a morphologic imaging test; size or shape of lymph node are the most important CT criteria for tumour involvement but they are limited by low sensitivity and specificity [63].

**Table 1:** Meta-analyses of mediastinal lymph node staging

Author	Sensitivity	Specificity	PPV	NPV	N Studies	N Pts
Fischer MB et al [57]	0,83	0.96	0,87	0,95	17	nr
Toloza EM et al [58]	0,84	0,89	0,79	0,93	18	1045
Helwing D et al [60]	0,88	0,92	nr	Nr	20	1292
Dwamena BA et al. [59]	0,79	0,91	0,9	0,93	16	639
Gould MK et al [61]	0,85	0,90	nr	Nr	32	1959
Birim O. et al [62]	0,83	0,92	nr	Nr	17	833

PPV: positive predictive value; NPV: Negative predictive value; Pts: Patients; nr: Not reported

On the one hand, of major clinical importance is the high negative predictive value of mediastinal FDG-PET (up to 97%)[47,64,65]. In Patients with mediastinal PET-negative results mediastinoscopy may be redundant and staging may be adequate without invasive procedure, proceeding directly to thoracotomy [18]. This led to the recommendation to omit mediastinoscopy in case of a negative mediastinal FDG-PET[64,66,67]

On the other hand, some conditions must be respected: sufficient FDG uptake in the primary tumor; use of a dedicated PET camera; absence of a central tumor or important hilar lymph node disease that may led to under-stage, obscuring coexisting N2 disease on PET [68,55,69]. Additionally, centrally located disease are also associated with a higher incidence of occult N2 disease than non-centrally located tumors [70].

Previous studies have reported that PET positive uptake in a hilar node is a risk factor for occult N2 disease [43,27,70]. In fact FDG-positive hilar nodes are predictive for microscopically (from a few cells to a few millimetres) involvement in mediastinal nodes. No other currently available imaging technique can detect such small volume disease.

As reported by Verhagen, FDG-PET reduces the number of mandatory mediastinoscopy procedures by 46% with no increase in unexpected N2 involvement at thoracotomy [27]. Even if PET seems superior in staging N2 disease, it is still unable to carefully differentiate N1 from N2 involvement. Notably, limits of PET consist of failure to show anatomic landmark and imperfect spatial resolution. These aspects could restrict its use to assess loco regional lymph node metastases [71,72]. The use of integrated PET-CT could attenuate this limit, improving anatomic localization of positive lymph nodes [73]. This could enable a better distinction between central tumours and contiguous lymph nodes (e.g. central tumour alone or central tumour with N2-disease), or between adjacent lymph node stations (e.g. hilar N1- or mediastinal N2-disease). Thus the simultaneous use of these two imaging exams may obtain greater staging accuracy than either test alone and so it should be constantly considered in the initial staging of lung cancer.[74]

In a cost-effectiveness analysis on use of PET for all N0 patients at CT scan, the results were encouraging. In fact the cost of PET scanning was almost counterbalanced by the more appropriate selection of patients for beneficial surgery [75].

On the contrary, the use of cervical mediastinoscopy non-selectively among stage 1 NSCLC has been reported to be not cost-effective [76] as the incidence of N2 disease is less than 3% [43].

Thus, on this latter prospective trial, Cerfolio et al suggest to reserve mediastinoscopy to only those patients with clinically staged N0 disease with a right upper lobe lesion (in which they found 10.4% positive rate for mediastinoscopy), and to limit EUS-FNA in patients staged N0 with tumors only in the right lower lobe (in which they found 15.4% positive rate for EUS-FNA). Moreover the authors recommend both mediastinoscopy and EUS-FNA in patients clinically staged as N1 after integrated PET/CT.

However, as described in a recent study by Al-Sarraf et al. [70] 16% of patients with negative uptake in mediastinum on integrated PET-CT had occult N2 disease following resection.

Other pathological factors such as tumor subtype, grade of differentiation, tumor size, T stage and SUV max of primary tumor tend to be associated with the biological aggressiveness of tumors [77] and are of prognostic significance rather than being predictors of occult metastases in mediastinal nodes [70].

In another study by Lee et al the maximum standardized uptake value is a predictor of individual lymph node metastasis in non-small cell lung cancer. Using SUV_max _of 5.3 to assign malignanc, y highly improve PET/CT accuracy, thus significantly reducing the number of false positive results. The authors conclude that in the absence of other indications for mediastinoscopy, (N1 disease, central or multiple tumors), mediastinoscopy can be omitted in patients who have a maxi-SUV of less than 5.3 in their N2 lymph nodes and low max-SUV primary tumor to mediastinal lymph node ratio, as found by integrated PET/CT analysis. [78]

Albeit the positive predictive value is plausible, it must not be forgotten that false positive results can be obtained in the case of anthracosilicosis, infection, or granulomatous disorders [26]. In these patients mediastinoscopy in mandatory to confirm N2 or N3 disease in order to ensure that patients with resectable N0 or N1 disease can gain the possibility of resolutive surgery.

Kuehl et al. have described a well defined PET/CT staging protocol. According to the authors the procedure should include the analysis of the chest and abdomen in order to stage locally and to detect distant metastases. Moreover the field should include the neck to detect supraclavicular lymph nodes involvement. [79]

However, the general recommendation is that if PET scan result is negative, invasive mediastionoscopy could be avoided; on the contrary, a positive PET scan, makes mediastinoscopy necessary for lymph node sampling because of possibility of false-positive PET scan [46].

Regarding the distant metastases detection, a recent randomized trial [55] suggests that the addition of PET scanning to a conventional workup identified more asymptomatic patients with distant metastases among those with suspected NSCLC. Moreover in several studies [52,64]. FDG-PET scan improved clinical staging of lung cancer patients. Unexpected extra thoracic metastases were detected by FDG-PET in 15% of patients without evidence of metastases after conventional staging. This could avoid futile mediastinoscopy and eventually thoracotomy.

#### Treatment

The use of PET can be applicable for selection or delineation of radiotherapy target volumes. In particular PET with 18F-FDG has taken on increasing importance for radiotherapy planning purposes. In fact because of the hight sensitivity of FDG PET for the staging of lymph node metastases in lung cancer implies that a negative PET examination could permit focusing on the primary neoplasm reducing target volumes [80].

Few studies have prospectively evaluated PET with 18 FDG in radiotherapy planning in term of its role of local control and survival. It has been reported that PET based mediastinal lymph nodes radiotherapy does not affect the local control reducing the target volume. [81]

Omitting elective nodal irradiation of clinically uninvolved nodal station is a way of reducing toxicity in non small cell lung cancer. [81] This modality could be successfully applied also for patients with limited disease small cell lung cancer (LD-SCLC) for whom the treatment of choice is concurrent chemo-radiotherapy. [82]

In particular in a study conducted on for 21 patients with N2/N3 NSCLC by van Der Wel et al. the use of PET/CT to define the radiotherapy target volume, demonstrated an increase in term of dose delivered to the tumour associated with a reduction of esophagus and lung toxicity. [83]

#### Response to Therapy

For assessing treatment with chemotherapy or radiotherapy in patients with non small cell lung cancer, PET and PET/CT play a major role than CT alone due to the fact that metabolism is a more sensitive marker for response to therapy than morphology. [84]

In patients undergoing therapy, imaging can play a crucial role and may aid in predicting the outcome of treatment regimens [85]. After radiotherapy, anatomic imaging alone has limited utility: fibrosis, atelecttasis or inflammatory infiltration related to radiation pneumonitis could hide residual tumour, thus tumour can be differentiated from scarring by using FDG PET [86]. CT has been shown to be suboptimal in restaging the mediastinum after therapy [87-89]. FDG PET is more sensitive than and as specific as traditional imaging to assess for residual disease or recurrence after intervention [90].

Dooms et al. in a very recent study evaluated the role of FDG PET in a particular subset of patients. This population was represented by resectable stage IIIA-N2 non small cell lung cancer subjects for whom therapy is often induction chemotherapy followed by resection. The authors concluded that FDG PET should select patients, after induction chemotherapy, who can be considered for surgical treatment [91]. In fact in a precedent study conducted by Rohren et al. were compared patients in which there were a residual FDG uptake after treatment with those ones without FDG uptake, with a poor prognosis in the first ones.[87]

Despite the results of the various treatments it needs to consider that the radiotherapy could be responsible of inflammatory reactions that could give false positive to FDG PET examination. For this reason, to have a real evaluation of the response to the treatment is necessary to attend some weeks after chemotherapy administration and several months after radiotherapy [92].

#### Follow-Up

The clinically efficacy of the follow-up of lung cancer and the treatment of recurrence are the subject of controversy[93]. Relapsing lung cancer might be considered as a poor prognostic factor and its diagnosis could have no therapeutic impact. On the other hand, local recurrence could be object of re-treatment with acceptable long term survival. In fact several studies have demonstrated that re-treatment after surgery may increase survival. Surgery as a second curative intent needs early diagnosis of relapse and appropriate selection of patients [94].

Thus another possible application of PET/CT is evaluation of patients with the suspect of recurrence during the follow-up. This is supported by clinical advantage to detect not only local relapse but also distant metastatic spreading, with a significant impact on patient's management and selection. [95]

When considering suspected recurrences, it has been reported that PET may have a major impact on treatment. In one study, PET affected 63% of patients with possible relapse [96].

In a recent study by Hellwig et al.[97] the authors state that FDG-PET accurately detects recurrent lung cancer, while the detection of unsuspected distant metastases avoids unnecessary treatments. They also affirm that SUV is an independent prognostic factor and that PET could help in the selection of patients who will benefit from surgical re-treatment.

Hence, also in this field, fusion technology, gaining anatomical and physiologic data will give useful information, supporting the correct management of these patients.

## Competing interests

The authors declare that they have no competing interests.
